# Interaction between parental psychosis and early motor development and the risk of schizophrenia in a general population birth cohort

**DOI:** 10.1016/j.eurpsy.2015.04.006

**Published:** 2015-09

**Authors:** E. Keskinen, A. Marttila, R. Marttila, P.B. Jones, G.K. Murray, K. Moilanen, H. Koivumaa-Honkanen, P. Mäki, M. Isohanni, E. Jääskeläinen, J. Miettunen

**Affiliations:** a Department of Psychiatry, Center for Clinical Neurosciences, University of Oulu, Oulu, Finland; b Medical Research Center of Oulu, Oulu University Hospital, University of Oulu, Oulu, Finland; c Center for Life Course Epidemiology and Systems Medicine, University of Oulu, Oulu, Finland; d Department of Psychiatry, University of Cambridge, Cambridge, UK; e Department of Psychiatry, Oulu University Hospital, Oulu, Finland; f Institute of Clinical Medicine, Psychiatry, University of Eastern Finland, Kuopio, Finland; g Department of Psychiatry, Kuopio University Hospital (KUH), Kuopio, Finland; h Department of Psychiatry, Lapland Hospital District, Rovaniemi, Finland; i Department of Psychiatry, Länsi-Pohja Healthcare District, Finland; j Department of Psychiatry, the Middle Ostrobothnia Central Hospital, Kiuru, Finland; k Mental Health Services, Joint Municipal Authority of Wellbeing in Raahe District, Finland; l Mental Health Services, Basic Health Care District of Kallio, Finland; m Visala Hospital, the Northern Ostrobothnia Hospital District, Finland

**Keywords:** Parental psychosis, Schizophrenia, Risk factor, Motor developmental milestone, Birth cohort

## Abstract

**Background:**

Delayed motor development in infancy and family history of psychosis are both associated with increased risk of schizophrenia, but their interaction is largely unstudied.

**Aim:**

To investigate the association of the age of achieving motor milestones and parental psychosis and their interaction in respect to risk of schizophrenia.

**Methods:**

We used data from the general population-based prospective Northern Finland Birth Cohort 1966 (*n* = 10,283). Developmental information of the cohort members was gathered during regular visits to Finnish child welfare clinics. Several registers were used to determine the diagnosis of schizophrenia among the cohort members and psychosis among the parents. Altogether 152 (1.5%) individuals had schizophrenia by the age of 46 years, with 23 (15.1%) of them having a parent with psychosis. Cox regression analysis was used in analyses.

**Results:**

Parental psychosis was associated (*P* < 0.05) with later achievement of holding the head up, grabbing an object, and walking without support. In the parental psychosis group, the risk for schizophrenia was increased if holding the head up (hazard ratio [HR]: 2.46; degrees of freedom [df] = 1; 95% confidence interval [95% CI]: 1.07–5.66) and touching the thumb with the index finger (HR: 1.84; df = 1; 95% CI: 1.11–3.06) was later. In the group without parental psychosis, a delay in the following milestones increased the risk of schizophrenia: standing without support and walking without support. Parental psychosis had an interaction with delayed touching thumb with index finger (HR: 1.87; df = 1; 95% CI: 1.08–3.25) when risk of schizophrenia was investigated.

**Conclusions:**

Parental psychosis was associated with achieving motor milestones later in infancy, particularly the milestones that appear early in a child's life. Parental psychosis and touching the thumb with the index finger had a significant interaction on risk of schizophrenia. Genetic risk for psychosis may interact with delayed development to raise future risk of schizophrenia, or delayed development may be a marker of other risk processes that interact with genetic liability to cause later schizophrenia.

## 1 Introduction

Schizophrenia is an etiologically heterogeneous syndrome caused by genetic and environmental factors. The onset of the illness is usually in the second or third decade of life but studies have found several observable sub-clinical signs of neuropathology in infancy, childhood and adolescence [29]. Family history of psychosis is well established, as a major risk factor for schizophrenia [28], [35], while one of the earliest documented developmental precursors is neuromotor dysfunction [8]. During the 1950s, Barbara Fish described children of mothers with schizophrenia to be at higher risk of pandysmaturation, considered as a marker for an inherited neurointegrative deficit in schizophrenia [8], [9]. Several high-risk studies have established that there are delays in the motor development of infants genetically at risk for schizophrenia [29], [8], [9], [25], [33], [23], [39], [10]. The findings of the general population studies support evidence from genetic high-risk research that delayed achievement of motor milestones [16], [46], [37], [43], [4], social deficiency [41] and later speech development [16] are robust risk factors for schizophrenia (see Supplementary data, Table S1).

Previous findings in the Northern Finland Birth Cohort 1966 (NFBC 1966) have provided further evidence of the association between motor delay and schizophrenia [13]. The main finding was that, a higher age for learning to stand, walk or become potty-trained was associated with increased occurrence of schizophrenia in adult life [13]. Later motor development correlated with poor school performance at the age of 16 years [14] and with later cognitive functioning in schizophrenia [45], [36]. It remains unknown whether motor delay and parental psychosis are independent risk factors for schizophrenia or whether they interact to confer risk; to the best of our knowledge, no previous prospective population-based study has examined this question. The aim of this study was to examine the age of achievement of motor milestones and parental psychosis and their interaction in respect to risk of schizophrenia. We hypothesized that parental psychosis would be associated with later achievement of motor milestones and that these two risk factors together would present a higher risk of schizophrenia than either of them alone.

## 2 Method

### 2.1 Sample

The present study is a prospective study using data from the Northern Finland Birth Cohort 1966 (NFBC 1966), which is a general population-based sample. It consists of 12,068 pregnant women and their 12,058 live-born children in the provinces of Lapland and Oulu. These births represent 96% of all births in this region in year 1966 [34]. The subjects have been followed up since mid-pregnancy until 2012. At the age of 16, 11,017 of the children were alive and living in Finland, but 84 refused permission to use their data. After excluding all twins (*n* = 258), the study sample consisted of 10,675 subjects. The Ethics Committee of the Northern Ostrobothnia Hospital District has given the written informed consent and approved the study design of the NFBC 1966 and keeps it under review.

### 2.2 Diagnosis of schizophrenia among the cohort members

The final sample (10,283) included 152 subjects with schizophrenia and 10,131 non-psychotic controls. Several sources of data were used to determine the diagnosis of schizophrenia in the cohort members (ICD-8/9: 295 except 295.7, ICD-10 F20, or DSM-III-R 295 except 295.7):

• the Finnish Hospital Discharge Register (FHDR) between years 1972–2012 (*n* = 127);
• hospital inpatient register (*n* = 112), outpatient registers: specialized health care between years 1998–2012 (*n* = 64) and primary health care between years 2011–2012 (*n* = 27);
• national registers of the Finnish Social Insurance Institute (sick days until the year 1999, disability pensions until the year 2000, reimbursable medications until the year 2006; *n* = 101);
• Finnish Center for Pensions until the year 2011 (*n* = 29);
• confirmation of the diagnosis in a subsample: hospital notes [27] and two follow-up interviews (*n* = 114, 75% of all schizophrenia diagnoses), at the ages of 34 years (between years 1999–2001) [19] and 43 years (between years 2008–2011).

See Supplementary data, Table S2 for further details on sources of schizophrenia diagnosis. Individuals with other non-organic psychotic diagnoses (*n* = 185) and mental retardation (*n* = 217) were excluded, including 10 cases with both; non-organic psychosis and mental retardation.

Finnish national registries have found to be very useful and reliable sources for case detection in schizophrenia [27], [31], [26].

### 2.3 Parental psychosis

Parental psychosis was defined as a parent (mother and/or father) suffering from non-organic psychosis (i.e. ICD-8: 295–299; ICD-9: 295, 2961E, 2962E, 2963E, 2964E, 2967, 297–299; ICD-10: F20, F22–F29) at any time between 1964–2005. Information of the parental psychosis was gathered from the FHDR (*n* = 526; 1972–2005), including outpatient registers from specialized care (1998–2005), and the disability pension register of the Finnish Center for Pensions (1964–2005; *n* = 43). In the present study, 5.5% (*n* = 569) of the study subjects had at least one parent with psychosis, and of them, 23 developed schizophrenia.

### 2.4 Motor developmental milestones

The information on motor, social and lingual development of the children was gathered in regular visits to the Finnish child welfare clinics by nurses and doctors interviewing the parents and observing the children during infancy and early childhood in monthly intervals [32]. This is a normal procedure in Finnish public health care and was not organized particularly for this study. The achievement times of each milestone, in months, were recorded on a separate welfare card. Before year 2007, the NFBC 1966 motor milestone information was a mixture of welfare card data on only walking and standing and parental responses to a questionnaire administered at 1 year of age [13], [14]. The previous milestone information was merged with completed welfare card data so that we included the new information in cases where the same cohort member had both; new and older information on milestone attainment. In the present study, only the age of achievement of motor milestone in the first year of life (in months) was investigated as motor skills have been commonly associated with schizophrenia risks, and as this data in NFBC 1966 is more complete and detailed than that of other milestones [16], [43], [4], [13], [14]. The following milestones were addressed: being able to hold the head up, to grab an object, to turn over from back to tummy, to sit without support, to touch the thumb with the index finger, to stand up, to stand without support and to walk without support.

### 2.5 Statistical methods

Student's *t*-tests were used to compare mean values of ages (months) of achieving milestones between those with and without parental psychosis. All the ages of reaching the milestones were normally distributed. Cox regression analysis was used to test the risk of schizophrenia by motor developmental milestones, parental psychosis and their interactions (parental psychosis × milestones). We set the level of statistical significance to *P* < 0.05. The results are reported as hazard ratios (HR) with 95% confidence intervals (95% CI) for a one-month delay in reaching each motor milestone. Times of emigration and death were used as censoring points in analyses. The sample included 317 (3.1%) deaths and 260 (2.5%) emigrants (information from the Population Register Center until 2011).

The covariates of the present study included gender, perinatal risk, antenatal maternal depression, family type and father's social class at the time of birth. Perinatal risk was a combined factor including any of the following: low gestational age (<37 weeks), low birth weight (≤2500 g) and perinatal brain damage [17]. Antenatal maternal depression (depressed/very depressed versus no depression) [20], family type (single-parent family versus two-parent family) [21] and father's social class (unskilled workers versus others) [21] were used as two-category variables.

After investigating every milestone variable separately, we scrutinized motor development as a whole in respect of schizophrenia by using a principal component analysis with one component model. The one principal component model explained 41.4% of the variation in the motor milestones, eigenvalue 3.3, communalities of the milestones varied between 0.34–0.76. The difference between parental psychosis groups and subsequent schizophrenia was tested with analysis of variance (ANOVA). All the statistical analyses were executed using SPSS 21.

### 2.6 The missing data

A total of 995 (9.7%) out of the 10,283 cohort members had no information on milestones. In different analyses, the amount of missing data regarding the milestones varied from 18.8 to 48.0%, except the variable “touching the thumb with the index finger”, which had 67.4% missing data. Among those with parental psychosis, missing data in general (information on any of the milestones available) was 10.4% and among those without parental psychosis 9.6%, and for the variable “touching the thumb with the index finger” 69.4% in parental psychosis group and 67.3% without parental psychosis. The missing data considering the information on any of the milestones available in those with subsequent schizophrenia was 9.9% and 9.7% in those without schizophrenia. For the variable “touching the thumb with the index finger” the amount of missing data was 67.1% in schizophrenia group and 67.4% without schizophrenia. The differences between proportions of missing data in above-mentioned groups were statistically non-significant. Therefore, the amount of missing data was not dependent on parental psychosis or subsequent schizophrenia. We also checked the proportion of missing data according to place of residence. Regarding the variable “touching the thumb with the index finger” the amount of missing data was higher in rural areas (*P* = 0.001). The missing data mainly resulted from some hospitals having discarded the records within time. The more complete information on variables walking and standing compared to other motor variables derived from 1-year questionnaire data, which was merged with new welfare card data.

## 3 Results

Altogether, 152 (1.5%) cohort members had schizophrenia by the age of 46. Of these, 23 (15.1%) had parents with psychosis compared to 546 (5.4%) of the non-psychotic cohort members. In general, parental psychosis was associated with increased risk of schizophrenia (hazard ratio [HR]: 3.14; degrees of freedom [df] = 1; 95% confidence interval [95% CI]: 2.02–4.89).

### 3.1 The associations between parental psychosis and developmental milestones

The cohort members with parental psychosis had, in general, a higher mean age of reaching each motor milestones compared to those without parental psychosis, except in turning from back to tummy. The difference was statistically significant in being able to hold the head up (*P* = 0.021), to grab an object (*P* = 0.026) and in walking without support (*P* < 0.001; Table 1).

### 3.2 The association between schizophrenia and developmental milestones

In the entire study population, later achievement of several early motor milestones was associated with an increased risk of schizophrenia: standing up (HR: 1.14; df = 1; 95% CI: 1.01–1.28), standing without support (HR: 1.22; df = 1; 95% CI: 1.08–1.37) and walking without support (HR: 1.15; df = 1; 95% CI: 1.09–1.21; Table 2).

### 3.3 The association between schizophrenia and developmental milestones by parental psychosis

When the group with parental psychosis was separately investigated, later achievement of the following motor milestones was associated with increased risk of schizophrenia: holding the head up (HR: 2.46; df = 1; 95% CI: 1.07–5.66) and touching the thumb with the index finger (HR: 1.84; df = 1; 95% CI: 1.11–3.06). In the group without parental psychosis, the following milestones increased the risk of schizophrenia: standing without support (HR: 1.21; df = 1; 95% CI: 1.06–1.39) and walking without support (HR: 1.22; df = 1; 95% CI: 1.10–1.37; Table 2).

### 3.4 Interactions between parental psychosis and delayed achievement of motor milestones in respect to risk of schizophrenia

When the risk of schizophrenia was investigated, parental psychosis had statistically significant interaction with the age of touching the thumb with the index finger (HR: 1.76; df = 1; 95% CI: 1.01–3.05; Table 2).

### 3.5 Covariates

After adjusting the results for gender and every other covariate separately and also in a fully adjusted model (gender, father's social class, perinatal risk, maternal antenatal depression and family type) the results generally stayed the same regarding parental psychosis groups and the total sample. The interaction between parental psychosis and touching the thumb with the index finger remained statistically significant when adjusting for every covariate separately and also in a fully adjusted model (Supplementary data, Table S3).

### 3.6 Principal component analysis results

**Fig. 1:**
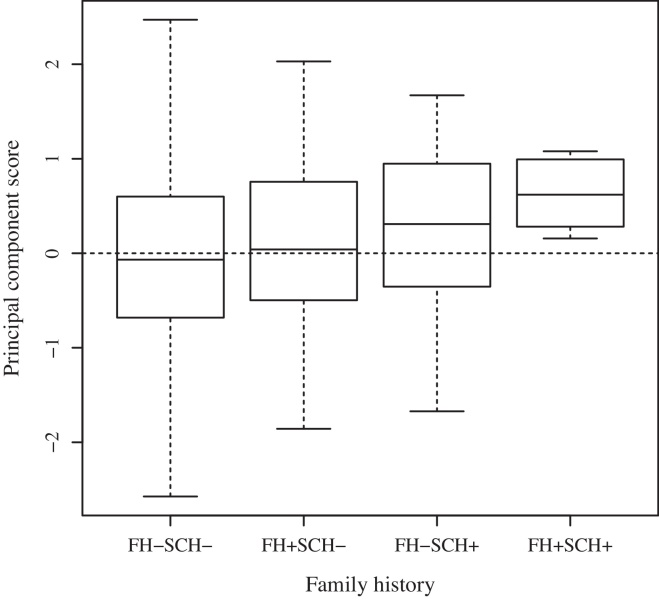
Principal component analysis was made to represent the motor development as a whole by single component. Fig. 1 shows that those with parental psychosis and subsequent schizophrenia develop the slowest. FH: family history of psychosis e.g. parental psychosis here; SCH: schizophrenia.

The results of the single principal component for motor development by parental psychosis are presented in Table 1 and further examined for subsequent schizophrenia in Table 2 and Fig. 1. See also Supplementary data, Table S4 for further details. Motor development as a whole was fastest in the group without parental psychosis and in those who did not develop schizophrenia and slowest in the group with parental psychosis and subsequent schizophrenia. However, the difference between groups was not statistically significant (F = 2.03; df = 3; *P* = 0.108). Additionally, we did not find interaction between parental psychosis and principal component in risk of schizophrenia (HR: 1.16; df = 1; 95% CI: 0.52–2.62; Table 2).

### 3.7 Results for other non-organic psychoses than schizophrenia, for schizophrenia spectrum disorders and for DSM-schizophrenia

When testing the association between parental psychosis and motor milestones in respect to other non-organic psychoses than schizophrenia, the pattern of the results was different. Motor milestones were not significant risks in parental risk group, but both in the total sample and in the group without parental psychosis, delayed standing and walking without support were significant risk factors for non-organic psychosis. The interaction between parental psychosis and touching the thumb with the index finger in the risk of non-organic psychosis was also significant (Supplementary data, Table S5).

In case of the schizophrenia spectrum disorders, the results, in general, were similar to those of schizophrenia (Supplementary data, Table S6). When those with DSM-schizophrenia (validated diagnoses) were separately analyzed and compared to those with any schizophrenia diagnosis (ICD + DSM), delayed walking without support was a significant risk for DSM-schizophrenia in all of the groups, while delayed touching the thumb with the index finger was a significant risk for schizophrenia only in the parental psychosis group. All other associations and also the interaction lost their significance (Supplementary data, Table S7).

## 4 Discussion

### 4.1 Main findings

Later achievement of motor milestones was associated with parental psychosis and the risk of schizophrenia. Parental psychosis was associated with delays in three of the eight milestones (i.e. holding-up the head, grabbing an object and walking without support). The risk of schizophrenia in the parental psychosis group was associated with later learning of holding-up the head, and touching the thumb with the index finger, while in the non-parental psychosis group the risk was associated with later ages of standing and walking without support. Moreover, parental psychosis had an interaction with touching the thumb with the index finger in respect to the risk of schizophrenia. Those with parental psychosis and subsequent schizophrenia had the slowest motor development compared to those without parental psychosis and subsequent schizophrenia. The amount of missing data considering the variable “touching the thumb with the index finger” was high but did not differ between parental psychosis groups or between those who develop schizophrenia and those who stay non-affected. The missing data mainly resulted from some hospitals having discarded the records already. Multiple analyses might have increased the risk of type 1 errors.

### 4.2 Comparison to earlier studies and theoretical discussion

#### 4.2.1 Parental psychosis and motor milestones

Those with parental psychosis achieved motor milestones later than those without parental psychosis; in particular, grabbing an object, holding the head up and walking without support were significantly later in this group.

In Fish's (1952) first description of pandysmaturation; the pan developmental deficit in neurological maturation and physical growth, she identified retarded infant motor development in the children of mothers with schizophrenia [8]. Weaker upper body muscle control, delays in reaching and picking up objects [23], in head control at the age of 4 months, and in walking with support [25], have been found in children of parents with schizophrenia. The trend was also similar in learning to sit, stand and walk without support [25]. These findings are consistent with our results and support the evidence of delays in both fine and gross motor development in the offspring of psychotic parents. Infants of parents with schizophrenia also have more neurological soft signs than infants of parents with no mental disorder [46].

#### 4.2.2 Milestones and risk of schizophrenia among the cohort members

In the entire study sample, we found three motor milestones in which delay increased the risk of schizophrenia (standing up, standing without support and walking without support). Previously, delay in walking has been the most significant risk factor of schizophrenia compared to other motor milestones in several studies [16], [43], [4], but delays in crawling, holding the head up, learning to sit, standing [43] and standing up have also increased the risk [4]. These findings were consistent with our results regarding the milestones standing up, standing without support and walking without support. Researchers have even been able to recognize those children who will develop schizophrenia from the healthy ones by observing their motor function [46] and social behavior [41] using home videos.

On the whole, our and earlier studies show that motor development in infancy is slightly later in those individuals who later develop schizophrenia compared to those who do not. This difference would not have been considered as abnormal in child welfare clinics.

#### 4.2.3 Parental psychosis, milestones and subsequent schizophrenia of the cohort members

In the parental psychosis group, the milestones achieved later (holding head up, touch thumb with index finger) were the milestones that appear earlier in a child's development than those in the group without parental psychosis (standing and walking without support) and in the total sample (standing up, standing and walking without support). To our knowledge, there have been no such results in previous studies.

In general, brain development is a complex sum of different mechanisms that occur in different times in the fetal period, infancy, childhood and adolescence. Genetic factors and brain self-produced internal activity have been found to play a substantial role in the first phase of brain development, whereas environmental conditions have not been found to have heavy influence in this phase. Environmental factors and epigenetic mechanisms have been shown to interact intensively in brain maturation later in the brain development [3]. These findings might explain our result that parental psychosis associates particularly to motor milestones that appear earlier in child's life when genetic factors play a key role in brain development.

#### 4.2.4 Interactions

To our knowledge, there are no other studies that have investigated the interaction between parental psychosis, delayed motor development and subsequent schizophrenia. We found one statistically significant interaction for parental psychosis. Touching the thumb with the index finger was a significant risk factor of schizophrenia only in the group with parental psychosis. This might mean that parental psychosis associates in particular to fine motor skills in respect of schizophrenia. The association between parental psychosis and fine motor skills has been shown before considering children at school-age [22], [6]. Although, some of the studies have found the opposite; gross motor skills have been seen to associate with parental psychosis [7]. Previously, we have found the interaction of parental psychosis in early biological and psychosocial, factors in respect to risk of schizophrenia [20], [18].

Parental psychosis might be either genetic, environmental or both. The risk associated with parental psychosis might be inherited within genes or result from environmental factors such as poor parental care or problems in mother child bonding. Both, genetic and environmental factors, may affect a child's development. Moreover, genetic effects are thought to interact with environmental risk factors by making genetically vulnerable individuals more sensitive to environmental stress effects [38], [47].

Environmental risk factors may affect a child's genes leading to mutations and epigenetic alterations such as histone acetylation and DNA methylation and subsequently affect brain development [5], [42]. Animal studies have found that variations in maternal care experienced during development profoundly impacts on the brain, altering stress reactivity, cognition and reproductive behavior [24]. Parental psychosis could be considered a source of chronic stress on the developing fetus and/or child, and as such could result in long-term neurobiological and architectural changes in the brain [1] for example via oxidative stress altering the function of HPA-axis [40]. In our study, all of the children with parental psychosis and subsequent schizophrenia were reared at home in the 1st year of life. Studies have also demonstrated that stressful experiences during the prenatal and postnatal periods may lead to subsequent dopamine dysfunction [15], [12], [30]: a potential mechanism of raising the risk of subsequent schizophrenia [11].

Twin studies have established that schizophrenia is highly heritable [2], [44]. Parental psychosis is considered as a marker of genetic risk for schizophrenia, but how might it interact with delayed development to raise the risk of subsequent schizophrenia? One possibility is that, delayed development is a marker of how genetic risk for schizophrenia operates. As parental psychosis is a risk factor for delayed development, some of the same genes that lead to delayed brain development also appear to confer risk of schizophrenia (operating via effects on neurodevelopment). Thus, observing delayed development in infancy can indicate that risk genes have been inherited and are taking an effect at an early age. It is shown that unaffected siblings of individuals with schizophrenia have more childhood neuromotor deficits, especially with motor coordination, than healthy controls. Researchers suggested that this can mark an inherited predisposition to schizophrenia and reflects the effect of genetic factors [37].

A further possibility is that delayed motor development may be a marker of other risk factors that interact with genetic risk, such as obstetric and perinatal factors. We recently showed that perinatal risk factors interact with parental psychosis to raise the risk of subsequent schizophrenia [18]. Thus, we considered the possibility that the interaction between motor development and parental psychosis might be secondary to delayed development being a marker of perinatal adverse events; however, as the interaction between touching the thumb with the index finger and parental psychosis persists despite adjusting for perinatal risk factors, this is unlikely to be the case.

### 4.3 Strengths

The NFBC 1966 is a prospective general population-based sample with high coverage and reliable sources of case findings, including Finnish national registries [26], which allow the use of data on deaths and emigration in the longitudinal analyses. Prospective information of mothers’ pregnancy and children's development has been possible to collect due to high coverage of Finnish maternity clinics and child welfare clinics. The quality of milestone information is now more accurate because of the whole welfare card data have been gathered by public health nurses and doctors. These assessments occurred in monthly intervals, as opposed to previous milestone data that frequently included parental responses measured only at 12 months. These factors allowed us to investigate the effects of risk factors of schizophrenia with adequate subsequent long-term follow-up.

### 4.4 Limitations

The FHDR only covers events since 1972, so there is a lack of information on hospital-treated parental psychoses at the time of, and before childbirth. This is a limitation primarily in those cases whose parents had schizophrenia but died before 1972 and were, therefore, not detected in our study. However, we have information on disability pensions from the year 1964, which should include most of the parents with psychosis. The diagnoses of hospital-treated severe mental disorders among the cohort members were validated until 1997 [27], while more recent (1998–2012) diagnoses of the cohort members and the diagnoses of parents were based on the clinical registers. Although the study sample is large with a relatively high number of subjects with schizophrenia, the subgroup analyses may lack statistical power. In this study we made multiple analyses, which increase the possibility of type 1 errors. All the milestones correlate with each other strongly and are therefore not independent risk factors. The amount of missing data is high regarding some of the variables, but did not differ between parental psychosis and subsequent schizophrenia groups.

## 5 Conclusions

Little previous research has investigated the interaction between parental psychosis and later achievement of motor developmental milestones on risk of schizophrenia. We found an interaction between parental psychosis and the age of touching the thumb with the index finger in infancy on risk of schizophrenia in the offspring. Parental psychosis was associated especially to motor milestones that appear early in child's life. Genetic risk for psychosis may interact with delayed development to raise future risk of schizophrenia, or delayed development may be a marker of other risk processes that interact with genetic liability to cause later schizophrenia. Further research is needed to clarify the complex etiology of schizophrenia in order to find ways to prevent schizophrenia in individuals with high risk.

## Disclosure of interest

The authors declare that they have no conflicts of interest concerning this article.

## Figures and Tables

**Table 1:** The association between parental psychosis and developmental milestones (in months).

	Parental psychosis		No parental psychosis		*t*^a^	df	*P*-value
	*n*	Mean (SD)	*n*	Mean (SD)			
Being able to hold head up when you lift their arms	292	2.23 (0.71)	5318	2.13 (0.74)	−2.32	5608	**0.021**
Make a grip on object (grab object)	290	3.32 (0.68)	5088	3.23 (0.69)	−2.23	5376	**0.026**
Turning from back to tummy	328	4.35 (1.19)	5716	4.37 (1.10)	0.27	6042	0.788
Sitting without support	279	7.33 (1.64)	5071	7.22 (1.13)	−1.12	293	0.265
Touch thumb with index finger (like a tweezer)	174	7.53 (1.22)	3175	7.40 (1.32)	−1.26	3347	0.206
Capable to stand up (lift themselves up)	292	8.58 (1.85)	5057	8.44 (1.38)	−1.27	310	0.206
Standing without support	440	10.48 (1.45)	7905	10.40 (1.34)	−1.20	8343	0.230
Walking without support	436	11.74 (2.15)	7459	11.46 (1.50)	−3.59	7893	**<0.001**
Principal component score	112	0.15 (1.03)	2173	−0.01 (0.99)	−1.67	2283	0.096

SD: standard deviation; df: degrees of freedom.

a Independent samples Student's *t*-test.

**Table 2:** The hazard ratios for developmental milestones (in months) and risk of schizophrenia in groups with and without parental psychosis and in the total study sample.

	Schizophrenia	Total		Parental psychosis			No parental psychosis			Interaction
		Mean (SD)	HR (95% CI)	*n*	Mean (SD)	HR (95% CI)	*n*	Mean (SD)	HR (95% CI)	HR (95% CI)
Holding the head up	No	2.13 (0.74)	Ref.	282	2.21 (0.71)	Ref.	5241	2.13 (0.74)	Ref.	Ref.
Yes	2.22 (0.78)	1.17 (0.89–1.54)	10	2.70 (0.68)	**2.46 (1.07–5.66)**	77	2.16 (0.78)	1.06 (0.78–1.42)	2.38 (0.98–5.79)

Gripping an object	No	3.24 (0.69)	Ref.	278	3.33 (0.68)	Ref.	5014	3.23 (0.69)	Ref.	Ref.
Yes	3.24 (0.59)	1.02 (0.75–1.38)	12	3.25 (0.45)	0.85 (0.36–2.02)	74	3.24 (0.62)	1.02 (0.74–1.42)	0.84 (0.34–2.10)

Turning from back to tummy	No	4.36 (1.10)	Ref.	315	4.35 (1.19)	Ref.	5639	4.36 (1.10)	Ref.	Ref.
Yes	4.52 (1.00)	1.13 (0.95–1.35)	13	4.38 (1.12)	1.02 (0.67–1.56)	77	4.55 (0.99)	1.16 (0.96–1.40)	0.88 (0.55–1.40)

Sitting without support	No	7.22 (1.16)	Ref.	266	7.33 (1.65)	Ref.	5006	7.22 (1.13)	Ref.	Ref.
Yes	7.32 (1.21)	1.06 (0.89–1.27)	13	7.31 (1.49)	0.99 (0.71–1.38)	65	7.32 (1.16)	1.08 (0.87–1.33)	0.92 (0.62–1.35)

Touching the thumb with the index finger	No	7.40 (1.31)	Ref.	167	7.49 (1.20)	Ref.	3132	7.40 (1.32)	Ref.	Ref.
Yes	7.64 (1.45)	1.15 (0.93–1.42)	7	8.57 (1.40)	**1.84 (1.11–3.06)**	43	7.49 (1.42)	1.05 (0.84–1.32)	**1.76 (1.01–3.05)**

Standing up	No	8.44 (1.41)	Ref.	277	8.56 (1.87)	Ref.	4991	8.43 (1.38)	Ref.	Ref.
Yes	8.75 (1.45)	**1.14 (1.01–1.28)**	15	8.87 (1.60)	1.05 (0.87–1.27)	66	8.73 (1.42)	1.16 (0.99–1.37)	0.90 (0.70–1.16)

Standing without support	No	10.40 (1.35)	Ref.	422	10.46 (1.46)	Ref.	7805	10.40 (1.34)	Ref.	Ref.
Yes	10.80 (1.36)	**1.22 (1.08–1.37)**	18	10.94 (1.16)	1.17 (0.93–1.48)	100	10.77 (1.40)	**1.21 (1.06–1.39)**	0.97 (0.74–1.27)

Walking without support	No	11.47 (1.54)	Ref.	418	11.70 (2.13)	Ref.	7370	11.46 (1.50)	Ref.	Ref.
Yes	12.13 (1.92)	**1.15 (1.09–1.21)**	18	12.67 (2.30)	1.08 (0.99–1.18)	89	12.02 (1.83)	**1.22 (1.10–1.37)**	0.88 (0.76–1.01)

Principal component score	No	0.00 (1.00)	Ref.	107	0.13 (1.04)	Ref.	2143	−0.01 (0.99)	Ref.	Ref.
Yes	0.31 (0.84)	1.33 (0.98–1.80)	5	0.63 (0.41)	1.48 (0.70–3.13)	30	0.26 (0.89)	1.28 (0.92–1.79)	1.16 (0.52–2.62)

SD: standard deviation; HR: hazard ratios for one-month delay, adjusted for gender.
