# Supplementary material for: Interaction between parental psychosis and early motor development and the risk of schizophrenia in a general population birth cohort
Source: Eur Psychiatry. 2015 Sep;30(6):719–27. doi: 10.1016/j.eurpsy.2015.04.006 (PMC4623356; doi:10.1016/j.eurpsy.2015.04.006)
Supplement: Supplementary file 1 [file mmc1.docx]

**Supplement Table 1.** The summary of the studies on motor milestones and schizophrenia.

| Authors (year) | Sample, Follow-up | Developmental milestones | Covariates | Results |
| --- | --- | --- | --- | --- |
| **GENERAL POPULATION** | | | | |
| Jones et al. (1994) [10]:  Births in England, Sadand, and Wales during the week March 3-9, 1946.  -Stratified random sample of 5362 individuals of a survey of 13 687. | - 30 individuals with schizophrenia. - 4716 Controls - Timing of early milestones was as recalled by the mother at age 2 | Milestones:  Sitting  Standing  Walking without support  Teething  Saying words (other than names of the parents) | - Sex - Social class | Risks for schizophrenia when learned late:   - Speech - Gross motor milestones - The greatest difference was for walking (p = 0.005) |
| Walker et al. (1994) [11]:  Part of the larger “Archival-Observational Project”  (N= 112) | - 30 schizophrenia subjects - 28 unaffected siblings - 21 controls - 19 affective disorder subjects and their 14 healthy siblings - Developmental data from observing home videos from birth to 2 years | - Neuromotor Rating Scale - Crawling - Grasping - Head control - Manual manipulation - Sitting - Walking | - Gender - Educational level | - Neuromotor abnormalities found in children with subsequent schizophrenia |
| Rosso et al. (2000) [12]:  The NCPP study  (N=8076) | - 72 individuals with schizophrenia of schizoaffective disorder - 63 unaffected siblings - 7941 controls - Examinations at 8 months, 4 and 7 years of age | Unusual movement  Motor coordination | - Gender - Race - Socioeconomic status - Parental education | - Motor coordination deficit and unusual movements associated to schizophrenia - Motor coordination deficits were more common in the unaffected siblings than in controls |
| Isohanni et al. (2001) [15]:  The Northern Finland birth cohort 1966.  (N=12058) | - 100 individuals with schizophrenia - 55 other psychoses - 315 non-psychotic disorders - 10 457 Controls - Developmental data from children’s visits to welfare centers and special 1-year examination - 31 year follow up | Milestones:  Standing without support  Walking without support  Potty-trained at age 1 year  Day/night wetting at age 1 year  Speaking at age 1 year.  Gross neurological development | - Sex - Maternal age at birth - Parity - Place of residence - Wantedness of the pregnancy - Social class | Risks for schizophrenia when learned late:   - Standing without support - Walking without support - Becoming potty-trained - Earlier milestones reduced, and later milestones increased, the risk in a linear manner compared with the whole cohort. |
| Sørensen et al. (2010) [13]:  the Copenhagen Perinatal cohort, born in 1959-1961.  (N=5765) | - 92 individuals with schizophrenia. - 691 individuals with other psychiatric disorder. - 4982 healthy cohort controls. - 45-year follow-up. - Developmental data were obtained from the mothers who filled up standardized diary to record the ages in weeks/months at which the child reached 12 developmental milestones | Milestones:  Smiling  Lifting head on stomach  Head holding when sitting  Grasping after things  Sitting without support  Rolling  Crawling  Crawling longer distance  Standing with/without support  Walking with/without support | - gender - mother’s age - father’s age - parental social status - breadwinner’s education - single mother status - parity | Risks for schizophrenia when learned late:   - Smiling - Lifting head - Sitting without support - Crawling - Walking without support |
| Schiffman et al. (2010) [16]:  Part of the larger study investigating the early signs of schizophrenia spectrum disorders  (N= 265) | - 16 schizophrenia subjects - 10 other psychotic disorders - 70 other psychiatric disorders - 146 controls - 81 individuals with parental schizophrenia | - At year 1972 when the study subjects were 11-13 years old their lunchtime meal habits were videotaped in highly standardized conditions. - Coding scheme for the videotapes was established. | - Gender - Socioeconomic status | - Those who later developed schizophrenia, had lower scores in sociability in childhood compared to those who stayed healthy and to those with other psychopathology. - Among males the score of general neuromotor scale differed significantly between schizophrenia group and other psychopathology group. |
| Clarke et al. (2011) [14]:  All individuals born in Helsinki between 1962 and 1969.  (N=378). | - 189 individuals with schizophrenia. - 189 healthy controls - Follow-up to year 2000. - Information source: child health card | Milestones:  Uttering first sound  Keeping head up  Grabbing an object  Turning over  Sitting unsupported  Pincer grip  Standing with and without support  Walking with and without support | - obstetric complications | Risks for schizophrenia when learned late:   - Sitting without support - Standing with and without support - Walking with and without support - Cumulative effect of developmental delay: with every additional delayed milestone, the odds of developing schizophrenia increased by 20% |
| **HIGH RISK STUDIES** | | | | |
| Mednick et al. (1971) [4]:  The Danish Obstetrical Study.  (N=249) | - 83 infants of parents with schizophrenia. - 83 infants of parents with character disorders. - 83 controls. - Examination at birth (1^st^ five days of life) and 1 year of age. | - Pediatricians examined the children at birth and at 1 year of life - At age 1 also mother’s reports | - Perinatal complications - The controls were matched by: - Gender of the parent and the child - Race - Multiple birth status - Birth order - Social class - Mother’s and father’s age - Mother’s height | - Children of parental schizophrenia had retarded motor development - Including delays in head control after 4 months and in walking with support - Trends toward delayed sitting, standing, and walking without support. |
| Ragins et al. (1975) [5]:  The Pittsburg Study.  (N=32) | - Infants of 14 mothers with schizophrenia. - 18 controls. - Examinations of the children and interviews of the mother. | - Study sample was divided into 3 groups and examined at different times (A: monthly during 2 weeks-3months and 3 months. B: between 4-8 months of age.   C: between 12-19 months of age.)   - Bayley scale assessments at 4-8 months and 12-19 months of age. | - Mother’s age - Race - Gender of the child - Birth complications - Maternal education - Family type - Multiple birth status | Association between parental schizophrenia and milestone:   - Developmental problems - Prehension skills |
| Marcus et al. (1981) [6]:  The Jerusalem Infant Study.  (N=58) | - 19 offspring with parental schizophrenia. - 20 offspring of parents with other psychiatric disorder. - 19 offspring from a control group | - Neonatal Behavioral Assessment Scale at 3 and 14 days of age. - Bayley Scales of Infant Development at 4, 8, and 12 months of age. - Carey Temperament Interview by the mothers at 4 and 8 months of age. - Re-interview for the family between 1 and 4 years. | - Perinatal complications - The controls were matched by: age, ethnic background, years of education, and gravidity. | Parental schizophrenia associated to:   - generally poor performance in motor skills during the 1^st^ year of life. |
| Sameroff et al. (1987) [7]:  The Rochester Longitudinal Study.  (N=184) | - Offspring of 29 mothers with schizophrenia. - 98 with parents with other psychiatric disorders. - 57 controls. - A 4-year longitudinal evaluation. | - The Bayley Scale at 4 and 12 months. - Cognitive, psychomotor, social, and emotional assessments at birth, 4, 12, 30, and 48 months of age. - The assessments were based on observing the mother and infant at the home and laboratory, and questionnaires. | - Socioeconomic status - Gender of the child - Family size | - The offspring with parental schizophrenia had significantly lower Bayley scores at 4 and 12 months. |
| Fish (1987, 1992) [3, 9]:  The New York Infant Study.  Births 1952-1953 and 1959-1960.  (N=24) | - 12 children of mothers with chronic schizophrenia - 12 controls from similar low socioeconomic status - Gesell testing and physical examinations 10 times between birth and 2 years of age - Follow-up until 1991 | Pandysmaturation (PDM):analysis of neurological maturation (Gesell’s test) and physical growth |  | - Pandysmaturation was related to being children of a mother with schizophrenia (P<0.05). - Pandysmaturation was also related to the severity of later psychiatric and cognitive disorder (P<0.01) at 10 years of age. |
| Erlenmeyer-Kimling et al. (2000) [31]:  New York High-Risk Project  (N=269) | - Infants of 79 parents with schizophrenia related psychoses - Infants of 57 parents with other psychiatric disorder - 133 infants of normal parents | - Lincoln-Oseretsky Motor Development Scale at 7-12 years of age |  | - Parental psychosis associated to deficits in gross motor skills - Deficits in gross motor skills associated to subsequent schizophrenia |

**Supplement table 2.** Sources for schizophrenia diagnoses in the NFBC 1966 (N=152).

|  | **Cases** | |
| --- | --- | --- |
| **Register** | **all** | **only** |
| **Finnish Hospital Discharge Register** | 127/152 (83.6%) |  |
| - Hospital inpatient (-2012) | 112/152 (73.7%) | 5/152 (3.3%) |
| - Primary health care outpatient (2011-2012) | 27/152 (17.8%) | 0 |
| - Specialized health care outpatient (1998-2012) | 64/152 (42.1%) | 0 |
| **Finnish Centre for Pensions (‑2011)** | 29/152 (19.1%) | 1/152 (0.7%) |
| **Social Insurance Institution** |  |  |
| - medicine (-2005) | 56/152 (36.8%) | 0 |
| - pension (-2000) | 31/152 (20.4%) | 0 |
| - sick days (-1999) | 14/152 (9.2%) | 0 |
| **Validation** | 114/152 (75.0%) | 25/152 (16.4%) |

**Supplement table 3.** The hazard ratios for developmental milestones (in months) and risk of schizophrenia in groups with and without parental psychosis and in the total study sample in a fully adjusted model.

|  |  | **Total** | | **Parental psychosis** | | | **No parental psychosis** | | | **Interaction** |
| --- | --- | --- | --- | --- | --- | --- | --- | --- | --- | --- |
|  | **Schizophrenia** | **mean (SD)** | **HR (95% CI)** | **N** | **mean (SD)** | **HR (95% CI)** | **N** | **mean (SD)** | **HR (95% CI)** | **HR (95% CI)** |
|  |  |  |  |  |  |  |  |  |  |  |
| Holding the head up | No | 2.13 (0.74) | Ref. | 282 | 2.21 (0.71) | Ref. | 5241 | 2.13 (0.74) | Ref. | Ref. |
|  | Yes | 2.22 (0.78) | 1.16 (0.88-1.55) | 10 | 2.70 (0.68) | **2.54 (1.07-6.01)** | 77 | 2.16 (0.78) | 1.05 (0.77-1.42) | 2.31 (0.95-5.62) |
|  |  |  |  |  |  |  |  |  |  |  |
| Gripping an object | No | 3.24 (0.69) | Ref. | 278 | 3.33 (0.68) | Ref. | 5014 | 3.23 (0.69) | Ref. | Ref. |
|  | Yes | 3.24 (0.59) | 1.04 (0.76-1.42) | 12 | 3.25 (0.45) | 0.86 (0.35-2.10) | 74 | 3.24 (0.62) | 1.05 (0.75-1.47) | 0.81 (0.32-2.04**)** |
|  |  |  |  |  |  |  |  |  |  |  |
| Turning from back to tummy | No | 4.36 (1.10) | Ref. | 315 | 4.35 (1.19) | Ref. | 5639 | 4.36 (1.10) | Ref. | Ref. |
|  | Yes | 4.52 (1.00) | 1.15 (0.96-1.37) | 13 | 4.38 (1.12) | 1.00 (0.65-1.53) | 77 | 4.55 (0.99) | 1.18 (0.97-1.43) | 0.86 (0.53-1.39**)** |
|  |  |  |  |  |  |  |  |  |  |  |
| Sitting without support | No | 7.22 (1.16) | Ref. | 266 | 7.33 (1.65) | Ref. | 5006 | 7.22 (1.13) | Ref. | Ref. |
|  | Yes | 7.32 (1.21) | 1.06 (0.88-1.26) | 13 | 7.31 (1.49) | 0.95 (0.67-1.36) | 65 | 7.32 (1.16) | 1.07 (0.86-1.32) | 0.92 (0.62-1.36) |
|  |  |  |  |  |  |  |  |  |  |  |
| Touching the thumb with the index finger | No | 7.40 (1.31) | Ref. | 167 | 7.49 (1.20) | Ref. | 3132 | 7.40 (1.32) | Ref. | Ref. |
|  | Yes | 7.64 (1.45) | 1.17 (0.94-1.45) | 7 | 8.57 (1.40) | **1.91 (1.05-3.47)** | 43 | 7.49 (1.42) | 1.08 (0.85-1.36) | **1.90 (1.03-3.48)** |
|  |  |  |  |  |  |  |  |  |  |  |
| Standing up | No | 8.44 (1.41) | Ref. | 277 | 8.56 (1.87) | Ref. | 4991 | 8.43 (1.38) | Ref. | Ref. |
|  | Yes | 8.75 (1.45) | **1.13 (1.00-1.28)** | 15 | 8.87 (1.60) | 1.03 (0.86-1.24) | 66 | 8.73 (1.42) | 1.16 (0.98-1.37) | 0.91 (0.71-1.17) |
|  |  |  |  |  |  |  |  |  |  |  |
| Standing without support | No | 10.40 (1.35) | Ref. | 422 | 10.46 (1.46) | Ref. | 7805 | 10.40 (1.34) | Ref. | Ref. |
|  | Yes | 10.80 (1.36) | **1.22 (1.08-1.38)** | 18 | 10.94 (1.16) | 1.15 (0.92-1.45) | 100 | 10.77 (1.40) | **1.22 (1.06-1.40)** | 0.96 (0.73-1.26) |
|  |  |  |  |  |  |  |  |  |  |  |
| Walking without support | No | 11.47 (1.54) | Ref. | 418 | 11.70 (2.13) | Ref. | 7370 | 11.46 (1.50) | Ref. | Ref. |
|  | Yes | 12.13 (1.92) | **1.14 (1.08-1.21)** | 18 | 12.67 (2.30) | 1.05 (0.96-1.15) | 89 | 12.02 (1.83) | **1.23 (1.10-1.38)** | 0.87 (0.76-1.01) |
|  |  |  |  |  |  |  |  |  |  |  |

SD=standard deviation. HR=Hazard Ratios for one month delay, adjusted for gender, perinatal risk, social class, family type and maternal antenatal depression

**Supplement table 4.** The correlation between motor milestone and principal component in the Principal component analysis.

|  | **Component 1*** |
| --- | --- |
| Walking without support | .791 |
| Standing without support | .821 |
| Standing up | .824 |
| Touching the thumb with the index finger | .576 |
| Sitting without support | .662 |
| Turning from back to tummy | .558 |
| Gripping on object | .379 |
| Holding head up | .327 |

*The one principal component model explained 41.4% of the variation in the motor milestones, eigenvalue 3.3, communalities of the milestones varied between 0.34-0.76.

**Supplement table 5.** The hazard ratios for developmental milestones (in months) and risk of any other psychosis than schizophrenia in groups with and without parental psychosis and in the total study sample.

|  |  | **Total** | | **Parental psychosis** | | | **No parental psychosis** | | | **Interaction** |
| --- | --- | --- | --- | --- | --- | --- | --- | --- | --- | --- |
|  | **Other psychosis** | **mean (SD)** | **HR (95% CI)** | **N** | **mean (SD)** | **HR (95% CI)** | **N** | **mean (SD)** | **HR (95% CI)** | **HR (95% CI)** |
|  |  |  |  |  |  |  |  |  |  |  |
| Holding the head up | No | 2.13 (0.74) | Ref. | 282 | 2.21 (0.71) | Ref. | 5241 | 2.13 (0.74) | Ref. | Ref. |
|  | Yes | 2.16 (0.71) | 1.06 (0.78-1.43) | 10 | 2.30 (0.82) | 1.23 (0.51-2.97) | 66 | 2.14 (0.70) | 1.02 (0.74-1.41) | 1.17 (0.47-2.93) |
|  |  |  |  |  |  |  |  |  |  |  |
| Gripping an object | No | 3.24 (0.69) | Ref. | 278 | 3.33 (0.68) | Ref. | 5014 | 3.23 (0.69) | Ref. | Ref. |
|  | Yes | 3.25 (0.79) | 1.04 (0.74-1.47) | 9 | 3.67 (0.71) | 1.93 (0.83-4.50) | 58 | 3.19  (0.78) | 0.92 (0.63-1.34) | 2.08 (0.83-5.22) |
|  |  |  |  |  |  |  |  |  |  |  |
| Turning from back to tummy | No | 4.36 (1.10) | Ref. | 315 | 4.35 (1.19) | Ref. | 5639 | 4.36 (1.10) | Ref. | Ref. |
|  | Yes | 4.41 (1.16) | 1.04 (0.86-1.25) | 11 | 4.36 (0.92) | 1.00 (0.62-1.61) | 75 | 4.41 (1.20) | 1.04 (0.85-1.28) | 0.96 (0.56-1.63) |
|  |  |  |  |  |  |  |  |  |  |  |
| Sitting without support | No | 7.22 (1.16) | Ref. | 266 | 7.33 (1.65) | Ref. | 5006 | 7.22 (1.13) | Ref. | Ref. |
|  | Yes | 7.27 (0.94) | 1.03 (0.85-1.24) | 12 | 7.08 (1.00) | 0.89 (0.56-1.41) | 63 | 7.30 (0.93) | 1.06 (0.86-1.31) | 0.84 (0.51-1.39) |
|  |  |  |  |  |  |  |  |  |  |  |
| Touching the thumb with the index finger | No | 7.40 (1.31) | Ref. | 167 | 7.49 (1.20) | Ref. | 3132 | 7.40 (1.32) | Ref. | Ref. |
|  | Yes | 7.46 (1.64) | 1.03 (0.80-1.32) | 5 | 6.40 (1.95) | 0.58 (0.34-1.00) | 32 | 7.63 (1.56) | 1.14 (0.87-1.49) | **0.50 (0.27-0.92)** |
|  |  |  |  |  |  |  |  |  |  |  |
| Standing up | No | 8.44 (1.41) | Ref. | 277 | 8.56 (1.87) | Ref. | 4991 | 8.43 (1.38) | Ref. | Ref. |
|  | Yes | 8.63 (1.78) | 1.09 (0.93-1.28) | 11 | 8.45 (1.57) | 0.96 (0.67-1.38) | 49 | 8.67 (1.83) | 1.12 (0.93-1.36) | 0.86 (0.57-1.29) |
|  |  |  |  |  |  |  |  |  |  |  |
| Standing without support | No | 10.40 (1.35) | Ref. | 422 | 10.46 (1.46) | Ref. | 7805 | 10.40 (1.34) | Ref. | Ref. |
|  | Yes | 10.67 (1.51) | **1.15 (1.02-1.29)** | 19 | 10.53 (1.43) | 1.03 (0.76-1.40) | 111 | 10.69 (1.53) | **1.17 (1.02-1.32)** | 0.88 (0.64-1.23) |
|  |  |  |  |  |  |  |  |  |  |  |
| Walking without support | No | 11.47 (1.54) | Ref. | 418 | 11.70 (2.13) | Ref. | 7370 | 11.46 (1.50) | Ref. | Ref. |
|  | Yes | 11.87 (1.99) | **1.12 (1.04-1.20)** | 20 | 11.55 (1.36) | 0.96 (0.74-1.25) | 102 | 11.93 (2.09) | **1.19 (1.07-1.32)** | 0.81 (0.61-1.08) |
|  |  |  |  |  |  |  |  |  |  |  |

SD=standard deviation. HR=Hazard Ratios for one month delay, adjusted for gender

**Supplement table 6.** The hazard ratios for developmental milestones (in months) and risk of schizophrenia spectrum disorders in groups with and without parental psychosis and in the total study sample.

|  |  | **Total** | | **Parental psychosis** | | | **No parental psychosis** | | | **Interaction** |
| --- | --- | --- | --- | --- | --- | --- | --- | --- | --- | --- |
|  | **Schizophrenia spectrum disorder** | **mean (SD)** | **HR (95% CI)** | **N** | **mean (SD)** | **HR (95% CI)** | **N** | **mean (SD)** | **HR (95% CI)** | **HR (95% CI)** |
|  |  |  |  |  |  |  |  |  |  |  |
| Holding the head up | No | 2.13 (0.74) | Ref. | 282 | 2.21 (0.71) | Ref. | 5241 | 2.13 (0.74) | Ref. | Ref. |
|  | Yes | 2.22 (0.80) | 1.17 (0.89-1.54) | 13 | 2.54 (0.78) | **2.46 (1.07-5.66)** | 92 | 2.17 (0.79) | 1.06 (0.78-1.42) | 2.38 (0.98-5.79) |
|  |  |  |  |  |  |  |  |  |  |  |
| Gripping an object | No | 3.24 (0.69) | Ref. | 278 | 3.33 (0.68) | Ref. | 5014 | 3.23 (0.69) | Ref. | Ref. |
|  | Yes | 3.23 (0.60) | 1.02 (0.75-1.38) | 15 | 3.33 (0.49) | 0.85 (0.36-2.02) | 87 | 3.21  (0.61) | 1.02 (0.74-1.42) | 0.84 (0.34-2.10) |
|  |  |  |  |  |  |  |  |  |  |  |
| Turning from back to tummy | No | 4.36 (1.10) | Ref. | 315 | 4.35 (1.19) | Ref. | 5639 | 4.36 (1.10) | Ref. | Ref. |
|  | Yes | 4.45 (1.09) | 1.13 (0.95-1.35) | 17 | 4.41 (1.06) | 1.02 (0.67-1.56) | 93 | 4.45 (1.10) | 1.16 (0.96-1.40) | 0.88 (0.55-1.40) |
|  |  |  |  |  |  |  |  |  |  |  |
| Sitting without support | No | 7.22 (1.16) | Ref. | 266 | 7.33 (1.65) | Ref. | 5006 | 7.22 (1.13) | Ref. | Ref. |
|  | Yes | 7.29 (1.19) | 1.06 (0.89-1.27) | 16 | 7.38 (1.46) | 0.99 (0.71-1.38) | 77 | 7.27 (1.13) | 1.08 (0.87-1.33) | 0.92 (0.62-1.35) |
|  |  |  |  |  |  |  |  |  |  |  |
| Touching the thumb with the index finger | No | 7.40 (1.31) | Ref. | 167 | 7.49 (1.20) | Ref. | 3132 | 7.40 (1.32) | Ref. | Ref. |
|  | Yes | 7.58 (1.52) | 1.15 (0.93-1.42) | 9 | 7.78 (2.22) | **1.84 (1.11-3.06)** | 48 | 7.54 (1.38) | 1.05 (0.84-1.32) | **1.76 (1.01-3.05)** |
|  |  |  |  |  |  |  |  |  |  |  |
| Standing up | No | 8.44 (1.41) | Ref. | 277 | 8.56 (1.87) | Ref. | 4991 | 8.43 (1.38) | Ref. | Ref. |
|  | Yes | 8.71 (1.44) | **1.14 (1.01-1.28)** | 18 | 8.72 (1.50) | 1.05 (0.87-1.27) | 77 | 8.70 (1.44) | 1.16 (0.99-1.37) | 0.90 (0.70-1.16) |
|  |  |  |  |  |  |  |  |  |  |  |
| Standing without support | No | 10.40 (1.35) | Ref. | 422 | 10.46 (1.46) | Ref. | 7805 | 10.40 (1.34) | Ref. | Ref. |
|  | Yes | 10.80 (1.32) | **1.22 (1.08-1.37)** | 23 | 10.96 (1.19) | 1.17 (0.93-1.48) | 125 | 10.71 (1.34) | **1.21 (1.06-1.39)** | 0.97 (0.74-1.27) |
|  |  |  |  |  |  |  |  |  |  |  |
| Walking without support | No | 11.47 (1.54) | Ref. | 418 | 11.70 (2.13) | Ref. | 7370 | 11.46 (1.50) | Ref. | Ref. |
|  | Yes | 12.11 (1.88) | **1.15 (1.09-1.21)** | 23 | 12.52 (2.13) | 1.08 (0.99-1.18) | 113 | 12.03 (1.83) | **1.22 (1.10-1.37)** | 0.88 (0.76-1.01) |
|  |  |  |  |  |  |  |  |  |  |  |

SD=standard deviation. HR=Hazard Ratios for one month delay, adjusted for gender

**Supplement table 7.** The hazard ratios for developmental milestones (in months) and risk of DSM-schizophrenia (n=114) in groups with and without parental psychosis and in the total study sample.

|  |  | **Total** | | **Parental psychosis** | | | **No parental psychosis** | | | **Interaction** |
| --- | --- | --- | --- | --- | --- | --- | --- | --- | --- | --- |
|  | **DSM-Schizophrenia** | **mean (SD)** | **HR (95% CI)** | **N** | **mean (SD)** | **HR (95% CI)** | **N** | **mean (SD)** | **HR (95% CI)** | **HR (95% CI)** |
|  |  |  |  |  |  |  |  |  |  |  |
| Holding the head up | No | 2.13 (0.74) | Ref. | 282 | 2.21 (0.71) | Ref. | 5241 | 2.13 (0.74) | Ref. | Ref. |
|  | Yes | 2.18 (0.80) | 1.10 (0.80-1.51) | 13 | 2.71 (0.76) | 2.46 (0.92-6.54) | 92 | 2.12 (0.79) | 1.00 (0.70-1.39) | 2.58 (0.91-7.35) |
|  |  |  |  |  |  |  |  |  |  |  |
| Gripping an object | No | 3.24 (0.69) | Ref. | 278 | 3.33 (0.68) | Ref. | 5014 | 3.23 (0.69) | Ref. | Ref. |
|  | Yes | 3.18 (0.61) | 0.89 (0.62-1.27) | 15 | 3.22 (0.44) | 0.79 (0.29-2.18) | 87 | 3.18  (0.63) | 0.89 (0.60-1.30) | 0.91 (0.31-2.65) |
|  |  |  |  |  |  |  |  |  |  |  |
| Turning from back to tummy | No | 4.36 (1.10) | Ref. | 315 | 4.35 (1.19) | Ref. | 5639 | 4.36 (1.10) | Ref. | Ref. |
|  | Yes | 4.54 (1.01) | 1.14 (0.94-1.40) | 17 | 4.63 (1.06) | 1.15 (0.75-1.76) | 93 | 4.53 (1.01) | 1.14 (0.91-1.42) | 1.00 (0.62-1.61) |
|  |  |  |  |  |  |  |  |  |  |  |
| Sitting without support | No | 7.22 (1.16) | Ref. | 266 | 7.33 (1.65) | Ref. | 5006 | 7.22 (1.13) | Ref. | Ref. |
|  | Yes | 7.33 (1.21) | 1.07 (0.87-1.31) | 16 | 7.33 (0.87) | 1.00 (0.68-1.48) | 77 | 7.33 (1.27) | 1.08 (0.85-1.38) | 0.92 (0.58-1.46) |
|  |  |  |  |  |  |  |  |  |  |  |
| Touching the thumb with the index finger | No | 7.40 (1.31) | Ref. | 167 | 7.49 (1.20) | Ref. | 3132 | 7.40 (1.32) | Ref. | Ref. |
|  | Yes | 7.68 (1.47) | 1.18 (0.92-1.51) | 9 | 8.59 (1.52) | **1.79 (1.02-3.13)** | 48 | 7.53 (1.44) | 1.08 (0.83-1.41) | 1.66 (0.90-3.06) |
|  |  |  |  |  |  |  |  |  |  |  |
| Standing up | No | 8.44 (1.41) | Ref. | 277 | 8.56 (1.87) | Ref. | 4991 | 8.43 (1.38) | Ref. | Ref. |
|  | Yes | 8.72 (1.50) | 1.13 (0.98-1.30) | 18 | 9.00 (1.76) | 1.07 (0.87-1.32) | 77 | 8.67 (1.45) | 1.13 (0.93-1.37) | 0.95 (0.72-1.26) |
|  |  |  |  |  |  |  |  |  |  |  |
| Standing without support | No | 10.40 (1.35) | Ref. | 422 | 10.46 (1.46) | Ref. | 7805 | 10.40 (1.34) | Ref. | Ref. |
|  | Yes | 10.67 (1.37) | 1.15 (1.00-1.34) | 23 | 11.00 (1.24) | 1.19 (0.93-1.54) | 125 | 10.61 (1.39) | 1.12 (0.95-1.33) | 1.07 (0.79-1.44) |
|  |  |  |  |  |  |  |  |  |  |  |
| Walking without support | No | 11.47 (1.54) | Ref. | 418 | 11.70 (2.13) | Ref. | 7370 | 11.46 (1.50) | Ref. | Ref. |
|  | Yes | 12.16 (2.08) | **1.15 (1.08-1.22)** | 23 | 13.00 (2.45) | **1.09 (1.00-1.19)** | 113 | 11.98 (1.96) | **1.21 (1.06-1.38)** | 0.90 (0.77-1.05) |
|  |  |  |  |  |  |  |  |  |  |  |

SD=standard deviation. HR=Hazard Ratios for one month delay, adjusted for gender

**Supplement table 8.** Demographics of the 152 individuals with schizophrenia.

| **152 INDIVIDUALS WITH SCHIZOPHRENIA** | |
| --- | --- |
| **Gender:** |  |
| Male | 93/152 (61.2%) |
| Female | 59/152 (38.8%) |
| **Parental psychosis:** |  |
| No | 129/152 (84.9%) |
| Yes | 23/152 (15.1%) |
| **Onsetage:** |  |
| Mean (minimun-maximum) | 27.0 (15.4- 46.3) years |
| **Diagnosis of Schizophrenia:** |  |
| ICD (register information) | 127/152 (83.6%) |
| ICD+DSM | 114/152 (75%) |
| DSM (only validated diagnoses until 1997) | 25/152 (16.4%) |

**Supplement table 9**. Correlation matrix for milestones, controls are above diagonal and individuals with schizophrenia are below the diagonal.

|  | Walking without support | Standing without support | Standing up | Touching the thumb with the index finger | Sitting without support | Turning from back to tummy | Gripping on object | Holding head up |
| --- | --- | --- | --- | --- | --- | --- | --- | --- |
| Walking without support | 1 | .755^**^ | .654^**^ | .287^**^ | .453^**^ | .335^**^ | .125^**^ | .100^**^ |
| Standing without support | .714^**^ | 1 | .649^**^ | .310^**^ | .436^**^ | .305^**^ | .137^**^ | .089^**^ |
| Standing up | .623^**^ | .682^**^ | 1 | .393^**^ | .572^**^ | .432^**^ | .172^**^ | .119^**^ |
| Touching the thumb with the index finger | ,154 | ,168 | .433^**^ | 1 | .414^**^ | .251^**^ | .231^**^ | .135^**^ |
| Sitting without support | .249^*^ | .460^**^ | .572^**^ | .482^**^ | 1 | .320^**^ | .166^**^ | .132^**^ |
| Turning from back to tummy | ,220 | .270^*^ | .407^**^ | .538^**^ | .334^**^ | 1 | .289^**^ | .186^**^ |
| Gripping on object | ,070 | ,202 | .249^*^ | ,250 | ,067 | .397^**^ | 1 | .423^**^ |
| Holding head up | ,092 | ,081 | ,127 | .323^*^ | ,201 | .309^**^ | .370^**^ | 1 |

**. Correlation is significant at the 0.01 level (2-tailed).
*. Correlation is significant at the 0.05 level (2-tailed).
